# Clinical efficacy of different surgical timing options in the treatment of congenital pediatric polydactyly

**DOI:** 10.12669/pjms.41.9.11636

**Published:** 2025-09

**Authors:** Xu Zhang, Nan Li, Jiani Qi, Shuai Han, Hongbian Zhu

**Affiliations:** 1 Xu Zhang Department of Paediatrics, Baoding Hospital, Beijing Children’s Hospital, Affiliated to Capital Medical University, Baoding 071000, Hebei, China; 2 Nan Li Department of Paediatrics, Baoding Hospital, Beijing Children’s Hospital, Affiliated to Capital Medical University, Baoding 071000, Hebei, China; 3 Jiani Qi Department of Paediatrics, Baoding Hospital, Beijing Children’s Hospital, Affiliated to Capital Medical University, Baoding 071000, Hebei, China; 4 Shuai Han Department of Paediatrics, Baoding Hospital, Beijing Children’s Hospital, Affiliated to Capital Medical University, Baoding 071000, Hebei, China; 5 Hongbian Zhu Department of Paediatrics, Baoding Hospital, Beijing Children’s Hospital, Affiliated to Capital Medical University, Baoding 071000, Hebei, China

**Keywords:** Congenital polydactyly, Efficacy, Joint function, Pediatric, Surgical timing

## Abstract

**Objective:**

To compare the clinical efficacy of different surgical timing options in the treatment of congenital pediatric polydactyly.

**Methods:**

A retrospective analysis was conducted on the clinical data of 50 children with congenital polydactyly treated surgically in Baoding Hospital, Beijing Children’s Hospital Affiliated to Capital Medical University from January 2020 to December 2023. According to the timing of surgery, they were divided into an early group (observation group, surgery < 3 years old) and a late group (control group, surgery >3 years old). The postoperative primary prognosis, incision healing time, clinical efficacy, satisfaction, and complications were compared between the two groups.

**Results:**

Both groups of children achieved primary healing, with a healing rate of 100.00% in both groups (all P>0.05). The postoperative active flexion angle of the interphalangeal and metacarpophalangeal joints in the observation group was higher than that in the control group (all P<0.05). The number of positive cases in the thumb adduction test and palm opposition test one month and three months postoperatively in the observation group was significantly higher than that in the control group (all P<0.05); however, the positive rates for thumb adduction and palm opposition function reached 100.00% in both groups six months and one year postoperatively (all P>0.05).

**Conclusion:**

Surgical intervention for congenital polydactyly in children before the age of three yields more favorable outcomes for postoperative finger shaping and functional recovery, while reducing the incidence of postoperative complications compared to surgery after the age of three.

## INTRODUCTION

Polydactyly, the most common congenital hand anomaly with an incidence ranging from 0.08% to 1.4%, predominantly affects the thumb, accounting for approximately 90% of cases.^1^,^2^ The condition is more prevalent in males than females, with a higher incidence in unilateral cases compared to bilateral ones, particularly on the right side. In China, statistical data indicate a male-to-female ratio of approximately 2:1 and a left-to-right ratio of about 1:1.5 for the condition.^3^ In contrast, international reports suggest a roughly equal male-to-female ratio and a similar distribution between the left and right sides, both averaging around 1:1.^4^ The etiology of polydactyly remains unclear, potentially linked to genetic inheritance and environmental changes. Given the critical role of finger dexterity in human manipulation abilities, polydactyly not only alters the appearance of the hand but also hinders functional development, leading to disability. Thumb polydactyly, in particular, can impair functions such as grasping, pinching, and abduction. Early diagnosis and treatment are therefore crucial to prevent functional damage to the fingers.

Despite this, there is no consensus on the optimal surgical timing for polydactyly. Traditionally, most experts have recommended surgery between the ages of four and six, when the finger anatomy is more clearly defined.^5^ Advances in technology and increased awareness of children’s psychological well-being have led some researchers in Europe and America to recommend earlier surgeries,^6^ ideally between six months and three years of age, considering this period crucial for the development of hand functions and minimizing psychological impact. The choice of surgical timing should, however, be tailored to the individual characteristics of Children. This study aimed to elucidate and investigate the surgical timing for congenital polydactyly in children, providing a clinical retrospective analysis to guide the selection of surgical timing for congenital pediatric polydactyly.

## METHODOLOGY

This retrospective study involved the selection of clinical data from 50 children with congenital polydactyly treated surgically in Baoding Hospital, Beijing Children’s Hospital Affiliated to Capital Medical University from January 2020 to December 2023. Based on the timing of surgery, children were divided into an early group (observation group, surgery before the age of three) and a late group (control group, surgery after the age of three).

### Ethical approval:

The study was approved by the Institutional Ethics Committee of Baoding Hospital, Beijing Children’s Hospital Affiliated to Capital Medical University (No.: 202335; Dated: April 03, 2023), and written informed consent was obtained from all participants’ parents or legal guardian.

### Inclusion criteria:

- Confirmed diagnosis of congenital polydactyly with radiographic evidence.
- Aged between six months and six years.
- Met surgical indications with complete clinical data.

### Exclusion criteria:

- Severe visceral dysfunction.
- Psychiatric disorders or speech impairments.
- Immune or hematologic diseases.
- Concomitant syndactyly.
- Incomplete clinical data.

All surgical procedures were conducted under general anesthesia, with a pneumatic tourniquet applied proximally to the upper arm. Targeted surgical techniques were employed based on the Wassel classification of the deformity. For Type-I deformities, a “Z” pattern incision was performed at the affected thumb, followed by subcutaneous dissection with ophthalmic scissors, with careful preservation of the collateral ligaments. The smaller thumb and extensor tendon were excised, and the collateral ligaments were sutured to the distal phalanx to reconstruct interphalangeal joint stability. The surgical approach for Type-II cases was similar to Type-I, involving the excision of one side of the duplicated thumb, skin grafting to cover the lateral skin incision of the thumb, and nail groove adjustment.

In Type-III cases, the dominant thumb was preserved, and the smaller thumb was excised. The procedure included a “Z” shaped incision, subcutaneous dissection, and preservation of collateral ligaments and the abductor pollicis brevis muscle. The smaller thumb and extensor tendon were removed, and the collateral ligaments were sutured to the base of the distal phalanx to stabilize the interphalangeal joint and reconstruct thumb abduction. For Types IV/V cases, the dominant thumb was preserved, and osteotomy was performed to correct the alignment of the carpal joint surfaces, preserving the extensor pollicis longus and flexor pollicis longus tendons of the excised thumb, which were transposed and sutured to the base of the terminal phalanx of the preserved thumb to correct the tendon alignment and ensure normal longitudinal force lines of the thumb. Types-VI and II followed similar principles, but joint capsule opening was required for the surgery. Type-VII surgeries were complex, generally preserving the dominant thumb, excising the smaller thumb, preserving collateral ligaments and abductor pollicis brevis for reconstruction, and performing osteotomy to correct abnormal force lines; if the preserved thumb was a triphalangeal thumb, the middle triangular bone segment was excised, fixed with Kirschner wires, and sutured.

### Postoperative care:

Dressings were changed on the second day post-surgery, with discharge occurring between 3-7 days. Patients undergoing soft tissue surgery were externally fixed for 3-4 weeks, while those undergoing osteotomy were fixed for 4-6 weeks. A follow-up outpatient visit was scheduled one postoperatively for the removal of external fixations. Parents were instructed to guide their children in using the operated hand to grasp toys and train thumb flexion-extension, opposition function, and wrist joint mobility, and follow-up for one year.

### Observation indicators:

The postoperative primary outcomes, incision healing time, joint function, thumb adduction and opposition ability, clinical efficacy, patient satisfaction, and incidence of complications were compared across all patients. Joint function was assessed using anatomical parameters that measured the limitations of active flexion and extension in the interphalangeal and metacarpophalangeal joints. Thumb adduction and opposition function were evaluated through the abductor pollicis muscle strength test and the thumb opposition test, respectively. Clinical efficacy was determined using the Kawabata scoring system, which included the range of motion of the joints, joint stability, and angular deformity. The scoring system categorized joint motion as follows: greater than 70° = 2 points, 50°-70° = One point, and less than 50° = 0 points. Stability was assessed with One point for stability and 0 points for instability. Angular deformity was scored as follows: less than 10° = 2 points, 10°-20° = One point, and greater than 20° = 0 points. The sum of the scores ranged from four to five for excellent outcomes, two to three for good outcomes, and 0 to One for poor outcomes. Satisfaction was gauged through functional and aesthetic aspects, with overall satisfaction calculated as (very satisfied + satisfied)/total number of cases × 100%. Postoperative complications included incisional infection, scar contracture, nail deformity, and secondary angular deformity.

### Statistical Analysis:

All data were statistically analyzed using the SPSS 22.0 software (SPSS Inc., Chicago, IL, USA). Categorical data were presented as n (%), and comparisons between groups were made using the c² test; measurement data were presented as mean±standard deviation (*X̄*±*S*), and comparisons between groups were made using independent samples t-test. A P-value <0.05 was considered a statistically significant difference.

## RESULTS

The control group consisted of 13 males and 12 females, aged between six months and six years, with deformity locations as follows: left hand in 12 cases, right hand in nine cases, and both hands in four cases. The Wassel classification was distributed as: Type-I in eight cases, Type-II in eight cases, Type-III in five cases, Type-IV in two cases, and Type-V in two cases, with 15 cases of polydactyly along the normal axis and 10 cases along a deviated axis. The observation group comprised 10 males and 15 females, aged between six months and seven years, with deformity locations as follows: left hand in 13 cases, right hand in seven cases, and both hands in five cases. The Wassel classification was distributed as: Type-I in nine cases, Type-II in seven cases, Type-III in six cases, Type-IV in two cases, and Type-V in one case, with 16 cases of polydactyly along the normal axis and nine cases along a deviated axis. No statistically significant difference was observed in the general data between the two groups (P>0.05).

All children in both groups achieved primary healing, with a healing rate of 100%. The comparison of hospital stays and wound healing time showed no statistically significant difference (P>0.05) Table-I. No statistically significant difference was observed between the two groups in terms of active extension angles of interphalangeal and metacarpophalangeal joints postoperatively (all P>0.05). However, the observation group exhibited significantly higher active flexion angles of interphalangeal and metacarpophalangeal joints than the control group (P<0.05) Table-II.

**Table-I:** Comparison of Primary Healing, Hospital Stay and Wound Healing Time Between the Two Groups.

Group	Primary Healing Rate	Hospital Stay (d)	Wound Healing Time (d)
Observation Group (n=25)	25 (100.00)	5.32±1.68	13.64±1.38
Control Group (n=25)	25 (100.00)	6.36±2.48	12.76±1.83
*χ*²/*t*	0.000	1.737	1.918
*P*	1.000	0.089	0.061

**Table-II:** Comparison of Postoperative Anatomical Parameters (*X̄*±*S*, °)

Group	Active Extension Angle of Interphalangeal Joints	Active Extension Angle of Metacarpophalangeal Joints	Active Flexion Angle of Interphalangeal Joints	Active Flexion Angle of Metacarpophalangeal Joints
Observation Group (n=25)	21.96±1.90	11.23±1.27	56.73±7.93	76.08±5.84
Control Group (n=25)	22.85±1.71	11.35±1.44	50.20±5.78	68.01±5.61
*t*	1.747	0.313	3.327	4.979
*P*	0.087	0.755	0.002	<0.001

The number of positive cases in thumb adduction and opposition tests one month and three months postoperatively was significantly higher in the observation group than in the control group, with a statistically significant difference (P<0.05). However, the positive rate of thumb adduction and opposition function reached 100% in both groups six months and one year postoperatively (P>0.05) Table-III.

**Table-III:** Comparison of Postoperative Thumb Adduction and Opposition Function [n(%)].

Time Point	Group	Adduction Test		Opposition Test	
		Positive	Negative	Positive	Negative
1 month	Observation Group (n=25)	6 (24.00)	19 (76.00)	8 (32.00)	17 (68.00)
	Control Group (n=25)	1 (4.00)	24 (96.00)	2 (8.00)	23 (92.00)
3 months	Observation Group (n=25)	12 (48.00)	13 (52.00)	13 (52.00)	12 (48.00)
	Control Group (n=25)	5 (20.00)	20 (80.00)	6 (24.00)	19 (76.00)
6 month	Observation Group (n=25)	25 (100.00)	0 (0.00)	25 (100.00)	0 (0.00)
	Control Group (n=25)	25 (100.00)	0 (0.00)	25 (100.00)	0 (0.00)
1 year	Observation Group (n=25)	25 (100.00)	0 (0.00)	25 (100.00)	0 (0.00)
	Control Group (n=25)	25 (100.00)	0 (0.00)	25 (100.00)	0 (0.00)

The postoperative range of motion in finger joints, joint stability, and efficacy scores in the observation group were superior to those in the control group, while the degree of angular deformity was lower than that in the control group, with statistically significant differences (all P<0.05) Table-IV. No statistically significant difference was observed in postoperative functional and aesthetic satisfaction between the two groups (all P>0.05) Table-V. The incidence of postoperative complications in the observation group was 8%, significantly lower than the 32% observed in the control group, with a statistically significant difference (c²=4.500, P<0.05) Table-VI.

**Table-IV:** Comparison of Postoperative Kawabata Scores (*X̄*±*S*, points).

Group	Range of Motion in Interphalangeal Joints	Joint Stability	Angular Deformity	Efficacy
Observation Group (n=25)	1.76±0.44	0.88±0.33	1.56±0.51	4.20±0.87
Control Group (n=25)	1.48±0.51	0.64±0.49	1.28±0.46	3.40±0.76
*t*	2.087	2.028	2.049	3.464
*P*	0.042	0.048	0.046	0.001

**Table-V:** Postoperative Satisfaction Comparison [n(%)].

Item	Group	Very Satisfied	Satisfied	Dissatisfied	Total Satisfaction
Function Satisfaction	Observation Group (n=25)	20 (80.00)	3 (12.00)	2 (8.00)	23 (92.00)
	Control Group (n=25)	18 (72.00)	4 (16.00)	3 (12.00)	22 (88.00)
Appearance Satisfaction	Observation Group (n=25)	21 (84.00)	3 (12.00)	1 (4.00)	24 (96.00)
	Control Group (n=25)	19 (76.00)	4 (16.00)	2 (8.00)	23 (92.00)

**Table-VI:** Comparison of Postoperative Complications [n(%)].

Group	Incision Infection	Nail Deformity	Secondary Angular Deformity	Scar Contracture	Total
Observation Group (n=25)	0 (0.00)	0 (0.00)	1 (4.00)	1 (4.00)	2 (8.00)
Control Group (n=25)	2 (8.00)	1 (4.00)	2 (8.00)	3 (12.00)	8 (32.00)
*χ*²					4.500
*P*					0.034

## DISCUSSION

Our findings reveal that, in contrast to the control group, the observation group exhibited higher active flexion angles in the interphalangeal and metacarpophalangeal joints post-surgery, with significantly higher positive cases in thumb adduction and opposition tests at one and three months postoperatively, and increased scores in joint range of motion, joint stability, and efficacy in the Kawabata scoring, while scores for angular deformity decreased. These results suggest that early surgery is more beneficial for improving joint function in children with congenital polydactyly. It is analyzed that young children undergo rapid growth and development. As they age, not only does the deformity become more severe, but the associated blood vessels, nerves, and tendons also differ from normal development.^7^ Furthermore, complex, unique, and compound cases of polydactyly often require multiple surgeries. Given that the functional capabilities of the hand are largely established by the age of two^8^,^9^, delaying surgery could impair hand function. Hence, early surgical treatment of polydactyly is recommended to mitigate its impact on skeletal growth and to alleviate psychological distress for both the child and their family.^10^ The level of satisfaction with the surgical outcomes of polydactyly is also a key indicator of surgical success. Our study indicates that the observation group had superior satisfaction with both the appearance and function post-surgery compared to the control group, although the difference was not statistically significant. The surgical management of polydactyly is governed by the principles of reconstructing both the function and appearance of the fingers. The procedure involves not only the removal of supernumerary digits but also the severance of abnormal tendinous connections, reconstruction of the joint capsule and collateral ligaments, correction of the axis, and restoration of joint flexibility.^11^,^12^

Polydactyly is one of the most common hand anomalies, with an incidence of approximately 0.05%-0.19% in newborns. Thumb polydactyly is the most prevalent type, accounting for about 90% of polydactyly cases. The exact etiology of polydactyly remains unclear but is thought to be closely associated with genetic factors, environmental pollution, viral infections, or a history of radiation exposure.^13^-^15^ The thumb, which contributes to 40% of the hand’s motor function, plays a crucial role. Thumb polydactyly not only severely impairs the motor function and normal development of the thumb, affecting grasping, pinching, and abduction movements but also causes significant inconvenience in daily life. Given its congenital nature, thumb polydactyly severely affects the aesthetic appearance of the hand, potentially impacting the child’s physical and mental health. Surgical intervention is therefore recommended for all cases of polydactyly, with the aim of removing supernumerary digits, correcting deformities, restoring the appearance of the fingers, improving joint mobility and stability, and ultimately enhancing the quality of life for the affected children.^16^ However, the optimal timing for surgical correction of polydactyly has not yet been universally agreed upon internationally.

Some studies^17^ suggest that the best timing for surgery is between six months and one year of age, based on the rationale that children at this age have not yet developed functional pinching or concerns about their self-image, and the risk associated with anesthesia is lower. Other theories propose that the ages of 4-6 years, when the anatomy of the fingers is more clearly distinguishable^18^, represent the optimal timing for surgery. With further research, many researchers have proposed that the age range of six months to three years is the most suitable for polydactyly surgery^19^, as this period is critical for the establishment of hand function, and the psychological impact of surgery on children is minimal. It is generally believed that surgery for polydactyly should be performed as early as possible, but the timing should be tailored to the individual case.^20^ Our study results indicate that surgery performed before the age of three yields more pronounced effects and a lower incidence of complications. This can be attributed to the stronger plasticity of children under three, allowing for a more natural and normal appearance of the fingers post-surgery and better improvement in grasping and mobility.

### Limitations:

It includes a limited number of cases and a brief follow-up period. Subsequent investigations with an expanded sample size and extended follow-up are required to further elucidate the clinical efficacy of various surgical timing options for the treatment of congenital pediatric polydactyly.

## CONCLUSIONS

While there is no marked discrepancy in satisfaction between surgery performed before and after the age of 3, early surgery is more conducive to the postoperative shaping and functional recovery of the fingers, and it significantly diminishes the occurrence of postoperative complications.

### Authors’ Contributions:

**XZ** and **NL:** Designed the study, drafted the manuscript, are responsible and accountable for the accuracy or integrity of the work.

**JQ** and **SH:** Performed the statistical analysis and involved in the writing of the manuscript.

**HZ:** Collected the data and participated in analysis. All authors read and approved the final manuscript.

## References

[1] Comer GC, Potter M, Ladd AL (2018). Polydactyly of the Hand. J Am Acad Orthop Surg.

[2] Thomas BP, Pallapati S (2020). Congenital thumb differences- current concepts. J Clin Orthop Trauma.

[3] Lin S, Tong K, Zhang G, Cao S, Zhong Z, Wang G (2020). Clinical Characteristics and Distribution of Thumb Polydactyly in South China:A Retrospective Analysis of 483 Hands. J Hand Surg Am.

[4] Wei X, Wang Z, Wei W, Rao Y, Yang J, Cheng Y (2022). Effect of operation timing on children with congenital thumb polydactyly. International Medicine and Health Guidance News.

[5] Kim JK, Al-Dhafer BAA, Shin YH, Joo HS (2021). Polydactyly of the thumb:a modification of the Wassel-Flatt classification. J Hand Surg Eur Vol.

[6] Iba K, Takayama S, Kawabata H, Horii E, Kazuki K (2020). Assessment of hand function using the functional dexterity test after opponensplasty in young children with Blauth type 2 hypoplastic thumb. J Pediatr Orthop B.

[7] Karaduman ZO, Turhal O, Turhan Y, Arıcan M, Güler C, Cangur S (2019). Comparison of Two Different Approaches to Treat a Hallux Valgus Deformity:Intramedullary Self-Locked Plates and Herbert Screws. Medicina (Kaunas).

[8] Khabyeh-Hasbani N, Tozzi D, Guerra SM, Koehler SM (2022). Radial Polydactyly. JBJS Rev.

[9] Hu CH, Thompson ER, Agel J, Bauer AS, Moeller AT, Novotny SA (2021). A Comparative Analysis of 150 Thumb Polydactyly Cases from the CoULD Registry Using the Wassel-Flatt, Rotterdam, and Chung Classifications. J Hand Surg Am.

[10] Álvarez LFG, Tenorio-Castaño J, Poletta FA, Santos-Simarro F, Arias P, Gallego N (2023). A large, ten-generation family with autosomal dominant preaxial polydactyly/triphalangeal thumb:Historical, clinical, genealogical, and molecular studies. Am J Med Genet A.

[11] Xu C, Yang X, Zhou H, Li Y, Xing C, Zhou T (2020). A novel ZRS variant causes preaxial polydactyly Type-I by increased sonic hedgehog expression in the developing limb bud. Genet Med.

[12] Xu J, Chen X, Teng X, Wang X, Chen H (2021). Complex radial polydactyly in a Chinese family:inclusion of triphalangism, triplication, and syndactyly. Ann Transl Med.

[13] McCarter JH, Zeledon RA, Cole SH, Layon SA, Nguyen JL (2023). Common Pediatric Hand Anomalies. Semin Plast Surg.

[14] Al-Qattan MM, Kattan AE, Al-Lazzam A, Gelidan AG (2017). A Modified Bilhaut-Cloquet Procedure for Zigzag Thumb Polydactyly Types III and IV. Plast Reconstr Surg Glob Open.

[15] Ullah S, Dasti JI, Malik S (2015). Descriptive epidemiology of hereditary musculoskeletal and limb defects in the isolated population of Chitral, North-West Pakistan. Pak J Med Sci.

[16] Ortiz-Cruz G, Luna-Muñoz L, Arteaga-Vázquez J, Mutchinick OM (2019). Isolated postaxial polydactyly:Epidemiologic characteristics from a multicenter birth defects study. Am J Med Genet A.

[17] Yao Y, Zhou H, Li L, Nan G (2021). Epidemiological statistics of congenital thumb duplication in the Chinese population. J Orthop Surg Res.

[18] Bjorklund KA, O'Brien M (2022). Local Anesthesia Alone for Postaxial Polydactyly Surgery in Infants. Hand (N Y).

[19] Nguyen JL, Ho CA (2022). Congenital Disorders of the Pediatric Thumb. JBJS Rev.

[20] Wessel LE, Daluiski A, Trehan SK (2020). Polydactyly a review and update of a common congenital hand difference. Curr Opin Pediatr.

